# Underutilization of Implantable Cardioverter Defibrillator in Primary Prevention of Sudden Cardiac Arrest

**DOI:** 10.4021/cr13w

**Published:** 2011-01-20

**Authors:** Umashankar Lakshmanadoss, Saadia Sherazi, Ashim Aggarwal, David Hsi, Mehmet K. Aktas, James P. Daubert, Abrar H Shah

**Affiliations:** a Division of Hospital Medicine, Johns Hopkins Bayview Medical Center, Johns Hopkins University, Baltimore, MD, USA; b Department of Internal Medicine, Unity Health System, Rochester, NY, USA; c Division of Cardiology, Unity Health System, Rochester, NY, USA; d Division of Cardiac Electrophysiology, University of Rochester, Rochester, NY, USA; e Division of Cardiac Electrophysiology, Duke University Medical Center, Durham, NC, USA

**Keywords:** Implantable cardioverter defibrillators, Sudden cardiac arrest, Underutilization

## Abstract

**Background:**

The aim of this study was to evaluate the overall use of implantable cardioverter defibrillators (ICD) for primary prevention of sudden cardiac arrest (SCA), among eligible patients from an outpatient cardiology clinic and to determine what factors might contribute to underutilization of ICDs.

**Methods:**

This report was a retrospective chart review of patients with ischemic or non-ischemic cardiomyopathy and left ventricular ejection fraction ≤ 35% from an outpatient cardiology practice from January 2005 to May 2008. These patients met the eligibility criteria for ICD implantation for primary prevention of SCA. A detailed review of medical records captured distribution of ICD implantation including future plans for ICD implant, patient preference against ICD use, presence of severe co-morbidities, and any other documented reasons/contraindications regarding ICD implantation.

**Results:**

Of the 275 patients who were eligible for ICD for primary prevention of SCA, 119 (43%) had an ICD implantation. ICDs were used in 84 (48%) eligible men and 35 (35%) eligible women (P 0.02). Among 156 (57%) patients who did not receive ICD, 79 (28%) had severe co-morbidities precluding them from having ICD. Twenty-six patients (10%) refused to have ICD implanted. The remaining 51 (19%) patient charts did not include any documentation regarding ICD use (future plan or contraindication).

**Conclusions:**

ICDs are underutilized for primary prevention of SCA, with rates of use being lowest among eligible women. This underutilization exists even after accounting for patient preferences and presence of severe co-morbid conditions that might make an otherwise eligible patient not a suitable candidate for ICD implantation.

## Introduction

Implantable cardioverter defibrillators (ICD) reduce the risk of sudden cardiac arrest (SCA) in a select group of patients [1-5]. ICDs are recommended for primary prevention of SCA, in patients with cardiomyopathy (ischemic or non-ischemic), left ventricular ejection fraction (LVEF) ≤ 35 %, and New York Heart Association (NYHA) Class II or Class III heart failure symptoms [6]. Previous studies have identified that ICDs are underutilized and gender/racial disparities exist in the use of ICDs among eligible patients who are hospitalized [7, 8]. Some of the factors that might contribute to these observed disparities include patient preferences against use of ICD, presence of co-morbidities that may be prohibitive for ICD implant, financial constraints and knowledge and attitudes of health care providers regarding ICD therapy. There is a need for better understanding of why these disparities exist. In this study we sought to determine the overall use of ICDs for primary prevention of SCD, in outpatient practice where the decision of ICD implantation will be taken mostly. Second, we explored factors that are associated with underutilization of ICDs and whether patient preferences and presence of severe co-morbidities play a significant role towards this observed underutilization.

## Patients and Methods

We retrospectively reviewed medical records of all patients from an outpatient general cardiology practice in Unity Health System, Rochester, New York from January 2005 to May 2008. Patients who were eligible for ICD for primary prevention of SCA were included in the study. Data was collected from active patient charts (patients who were consulted at least once in the previous one year). Patients with history of ischemic or non-ischemic cardiomyopathy, LVEF ≤ 35% and NYHA Class II-III heart failure symptoms were considered eligible for an ICD implantation for primary prevention of SCA. LVEF was measured by echocardiography, nuclear myocardial perfusion imaging and/or by left ventricular angiogram. Patients with myocardial infarction in the past 40 days, coronary revascularization in the past 3 months and diagnosis of non-ischemic cardiomyopathy within last 9 months were excluded, as per the established guidelines [6].

Patient’s demographics and clinical data including age, gender, race, etiology and duration of cardiomyopathy, NYHA functional class, left ventricular ejection fraction and medications were recorded. The outcome measure of interest was overall use of ICDs among eligible patients including any future plan for ICD implant. We also looked for any documentation regarding patients’ own preference against the use of ICD and co-morbidities with life expectancy less than 1 year or overall poor functional status. The Institutional Review Board of Unity Health System approved the study protocol.

Categorical variables are presented as percentages and continuous variables are presented as mean with standard deviations. We used Chi-square for categorical variables and Student’s t-test for continuous variables to compare the baseline clinical characteristics of patients who received ICD with those who did not receive ICD implantation. Multivariate logistic regression analysis was used to identify important factors associated with ICD use among eligible patients. All analyses were performed using SAS software version 9.2. A P value of less than 0.05 was considered statistically significant.

## Results

Total of 10,254 patients’ medical records were reviewed. There were 275 patients who met the eligibility criteria for ICD implantation and 119 (43%) had ICD implanted. There were 35 (35%) female patients who received ICD and 84 (48%) male patients who underwent ICD implantation (P = 0.02). Twenty-six (10%) patients declined to receive an ICD. More women 15 (15%) refused an ICD implant than men 11 (6%) (P = 0.01). Baseline demographics and clinical characteristics of all ICD eligible patients are shown in Table 1.

**Table 1:** Baseline Characteristics of Patients Who Received ICD vs. Who Did Not Received ICD

	Total (275)	No ICD	Yes ICD	P value
Age (mean ± SD) in yr	73.4 ± 13.1	73.12 ± 14.0	73.86 ± 11.9	0.64
Gender (women)	101 (37%)	66 (65)	35 (35)	0.02
Race (Caucasian)	236 (86%)	132 (56)	104 (44)	0.5
LVEF	24.09 ± 6.72	23.7 ± 7.05	24.5 ± 6.2	0.35
LVEF ≤ 30%	243 (88%)	138 (57)	105 (43)	0.95
Cardiomyopathy				
Ischemic	228 (83%)	127 (56)	101 (44)	0.44
Non-Ischemic	47 (17%)	29 (62)	18 (38)	
ACE-I/ARB	233 (86%)	128 (55)	105 (45)	0.25
Statin	202 (75%)	113 (56)	89 (44)	0.98
Aspirin	200 (76%)	114 (57)	86 (43)	0.74
Beta blocker	255 (94%)	145 (57)	110 (43)	0.29
Serum BUN (in mgs%)	27.61 ± 14.25	27.4 ± 15.6	27.8 ± 12.2	0.79
Serum Creatinine (in mgs%)	1.35 ± 0.96	1.41 ± 1.21	1.26 ± 0.43	0.19

ICD: Implantable Cardioverter Defibrillator

LVEF: Left Ventricular Ejection Fraction

ACE-I: Angiotensin Converting Enzyme Inhibitor

ARB: Angiotensin Receptor Blocker

The mean age of patients who received ICD was 74 ± 12 years and mean age for patients who did not receive ICD was 73 ± 14 (P = 0.64). The ICD was implanted among 104 (55%) Caucasian patients and 15 (39%) African American patients (P = 0.59). There was no difference in ICD use in patients with ischemic cardiomyopathy vs. those with non-ischemic cardiomyopathy (P = 0.44) and in patients with LVEF ≤ 30% vs. those with LVEF 31% - 35% (P = 0.61). There was no difference in ICD use in the group of patients with age < 70 when compared with patients ≥70 years (P = 0.21). Similarly, there was no difference in ICD use among patients < 80 years compared with patients ≥ 80 years (P = 0.56). The other measures of quality of care such as use of angiotensin-converting enzyme inhibitor/angiotensin receptor blockers and beta blockers was similar in patients with ICDs as compared with those without ICD therapy.

**Figure 1:**
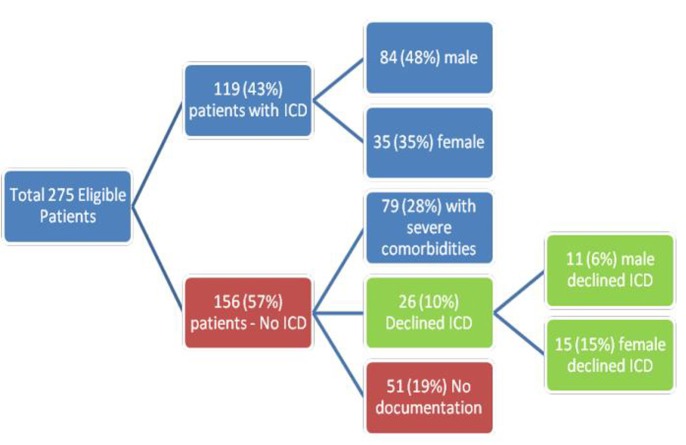
Utilization of implantable cardioverter defibrillator for primary prevention of sudden cardiac arrest. ICD: Implantable Cardioverter Defibrillator.

Of the 156 patients who did not receive an ICD, potential contraindications/reasons against ICD use were documented in 79 patients. These reasons included: co-morbidities (advanced dementia, malignancy with less than 1 year of life expectancy, acquired immune deficiency syndrome, advanced chronic kidney disease, advanced congestive heart failure with hospice care) and advanced age > 90 years. Twenty-six (10%) patients declined to receive an ICD. More women 15 (15%) refused an ICD implant than men 11 (6%) (P = 0.01). The distribution of ICD in this study population is shown in Figure 1.

**Figure 2:**
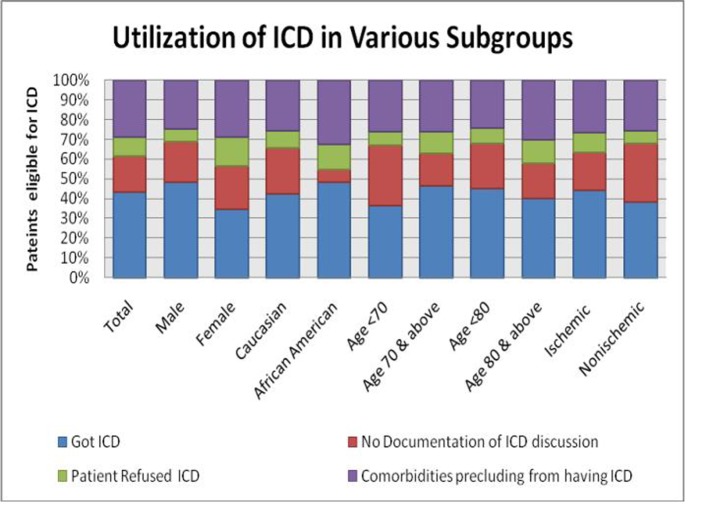
Utilization of ICD in various subgroups.

For the remaining 51 patients who did not receive an ICD, there was no documentation regarding future plan for ICD implant or potential contraindication for ICD use. In multivariable logistic regression model including age, gender, race, etiology of cardiomyopathy (ischemic vs. non-ischemic cardiomyopathy) and LVEF (≤ 30 vs. 31% - 35%), male gender was the only factor associated with use of ICD (OR 1.88; 95% CI 1.03 - 3.4 P 0.03). A detailed distribution of ICD utilization, co-morbid conditions, and refusal by gender is shown in Figure 2.

## Discussion

Our study describes an overall underutilization of ICDs (43%) for primary prevention of SCA in patients followed in outpatient cardiology clinic. The rates of ICD use were significantly lower (35%) among eligible women. There were 26 (10%) patients who made the choice not to have an ICD implantation. Interestingly there were more women who refused ICD implant as compared with men (15 % vs 6%, P = 0.01). The presence of co-morbid conditions including dementia, malignancy, and advance age played a significant role in the decision making for ICD implantation.

Recent literature describes underutilization of ICDs in patients hospitalized for heart failure [8]. In this study, we collected data from patients who were seen in outpatient cardiology setting and were eligible for ICD for primary prevention of SCA. This report offers a better understanding of ICD use beyond hospital admission for heart failure as in routine clinical practice, ICDs are not usually implanted in acute hospital admission for heart failure. The ICD use was significantly lower among women who also had higher refusal rate for ICD implant. Although there have been several factors proposed that could explain lower use of ICD among women, it remains unknown why they had higher refusal rates [7, 8]. The larger clinical trials evaluating the effectiveness of ICDs had lower participation of women (8% - 29%), thus limiting the generalizability of findings for women and potentially affecting the physician’s referral for ICD implantation in this group of patients [9]. A recent meta-analysis reported that ICD therapy for primary prevention of SCD in women did not reduce all-cause mortality [9]. Peterson et al reported that women had more adverse events related to ICD implantation than men [10]. These factors may also contribute to the lower rates of ICD referral among women. In a recent survey of physicians including cardiologists and primary care physicians, 27% participants were unsure regarding benefits of ICDs in eligible women and African Americans [11]. It is also plausible that women’s higher refusal rate could be related to more concerns regarding quality of life and possible cosmetic concerns resulting from ICD implantation. The ACC/AHA guidelines recommend ICD use for all patients regardless of their gender [6]. Further research is needed to explore reasons to why more women preferred against ICD implantation.

Although prior studies have shown disparity in ICD use among various racial/ethnic groups, we did not find any differences in the ICD implantation rate in Caucasians vs. non-Caucasians [7, 8]. Similarly, there were no differences in ICD use among patients of various age groups. This is encouraging, as with increase in aging population physicians are more likely to see a higher number of elderly patients. Furthermore, current literature supports the use of ICD in older patients [12]. However, presence of co-morbid conditions and quality of life should be considered in the decision making process. In our report, ICD treatment was deferred appropriately in patients with significant advanced diseases and poor prognosis. We did not find any differences in the use of ICDs among patients who have ischemic or non-ischemic cardiomyopathy as there is sufficient evidence now that suggests the ICD therapy is effective in SCD prevention in both conditions [13].

Our study has certain limitations including a small sample size and data from single cardiology practice. Therefore, the results may not be generalized to all patient populations. In addition, although documentation for a small proportion of patients was lacking, it does not completely exclude the possibility of a potential contraindication for ICD use. Follow up data for the patients who did not get their ICD was not available.

In conclusion, there is an underutilization of ICD among eligible patients who are seen in outpatient cardiology office even after accounting for patient preferences and presence of co-morbid conditions. The use of ICD is significantly lower among women who also have higher refusal rate to receive an ICD. Future research is needed to better understand reasons for patient’s preferences against ICD use especially among women.

## Abbreviations

ICD: Implantable Cardioverter Defibrillator
SCA: Sudden Cardiac Arrest
LVEF: Left Ventricular Ejection Fraction
NYHA: New York Heart Association
